# Supplementary material for: Allosteric regulation and crystallographic fragment screening of SARS-CoV-2 NSP15 endoribonuclease
Source: Nucleic Acids Res. 2023 Apr 28;51(10):5255–70. doi: 10.1093/nar/gkad314 (PMC10250223; doi:10.1093/nar/gkad314)
Supplement: gkad314_Supplemental_Files [file gkad314_supplemental_files.zip › Supplementary datasets.zip!/134/Refine_9/01-BUSTER/Cycle-3/shell.01/LogLik0.html]

```
# <Log-Likelihood> of null-hypothesis


<Log-Likelihood of null hypothesis>, 49168 all data           :      -0.88248

<Log-Likelihood of null hypothesis>,  2531 data in free set   :      -0.97533

<Log-Likelihood of null hypothesis>, 46637 data in working set:      -0.87744

```

```
```
